# Severe Refractory Hypotension Following Patent Blue V Injection: A Case of Atypical Perioperative Anaphylaxis

**DOI:** 10.3390/reports9030295

**Published:** 2026-09-02

**Authors:** Francesco Russano, Vittoria Patti, Cinzia Bettin, Massimiliano Caravello, Davide Brugnolo, Paolo Del Fiore

**Affiliations:** 1 Soft-Tissue, Peritoneum and Melanoma Surgical Oncology Unit, Veneto Institute of Oncology IOV-IRCCS, 35128 Padua, Italy; francesco.russano@iov.veneto.it; 2 Department of Surgery, Oncology and Gastroenterology (DISCOG), University of Padua, 35122 Padua, Italy; vittoria.patti@iov.veneto.it (V.P.); davide.brugnolo@iov.veneto.it (D.B.); 3 Anesthesiology Unit, Veneto Institute of Oncology IOV-IRCCS, 35128 Padua, Italy; cinzia.bettin@iov.veneto.it (C.B.); massimiliano.caravello@iov.veneto.it (M.C.)

**Keywords:** Patent Blue V, perioperative anaphylaxis, sentinel lymph node biopsy, melanoma, refractory hypotension, serum tryptase, intradermal testing, case report

## Abstract

**Background and Clinical Significance**: Patent Blue V (PBV) is widely used for sentinel lymph node mapping but may rarely cause severe hypersensitivity reactions. These reactions can present atypically, with predominant cardiovascular collapse and minimal cutaneous manifestations, potentially delaying recognition and treatment. **Case Presentation**: We report the case of a 52-year-old woman who developed sudden and prolonged refractory hypotension shortly after PBV injection during melanoma surgery. The reaction initially occurred in the absence of bronchospasm or early significant cutaneous manifestations. Profound cardiovascular collapse preceded the delayed appearance of erythema and piloerection, making early recognition of perioperative anaphylaxis particularly challenging. Hemodynamic instability required aggressive fluid resuscitation and prolonged vasopressor support. Subsequent diagnostic work-up, including serum tryptase measurement and allergological evaluation, confirmed PBV-induced anaphylaxis. PBV-induced anaphylaxis may present with isolated cardiovascular collapse without typical clinical features. **Conclusions**: Early recognition and prompt hemodynamic support are essential to optimize outcomes in the perioperative setting.

## 1. Introduction and Clinical Significance

Perioperative hypersensitivity reactions are most commonly triggered by neuromuscular blocking agents (70%), antibiotics, and latex (10%); however, emerging causative agents have been increasingly recognized in recent years [1,2]. Among these, hypersensitivity reactions to Patent Blue V (PBV) dye have been increasingly reported [3,4,5,6,7,8]. PBV dye finds its application in the identification of sentinel lymph nodes in patients with melanoma, breast cancer and other malignancies [9]. PBV-induced reactions are thought to be predominantly IgE-mediated, although the exact immunological mechanism remains incompletely understood and additional pathways have been proposed.

Immediate hypersensitivity reactions to Patent Blue V have been classified into four severity grades. Across published series, the reported frequencies are approximately as follows: Grade 1 (69–87%): urticaria, pruritus, blue wheals and generalized rash; Grade 2 (3.2–8%): transient hypotension (systolic blood pressure < 70 mmHg) without the need for vasopressors and/or bronchospasm/laryngospasm; Grade 3 (1.1%): severe cardiovascular collapse requiring vasopressors and/or interruption of the planned procedure and/or admission to the intensive care unit; and Grade 4 (<1%): respiratory or cardiorespiratory failure [2].

Here, we report the case of a 52-year-old woman with lumbar and right shoulder melanoma who developed a life-threatening anaphylactic reaction following Patent Blue V injection, characterized by severe cardiovascular collapse with delayed onset of cutaneous manifestations. The patient had no previous exposure to Patent Blue V and initially presented without cutaneous manifestations or bronchospasm. Cutaneous signs developed only after the onset of severe cardiovascular collapse, making the early recognition and management of perioperative anaphylaxis particularly challenging.

## 2. Case Presentation

A 52-year-old woman with right shoulder and lumbar melanoma was scheduled for sentinel lymph node biopsy and wide local excision. She had no history of drug allergies and had previously undergone three surgical procedures without complications. No prior medical exposure to Patent Blue V was documented.

A detailed perioperative timeline, including hemodynamic parameters, clinical manifestations, and therapeutic interventions, is reported in Table 1. The drugs administered during the perioperative and intensive care management are summarized in Table 2.

After mild sedation we administered in the lumbar scar’s dermal layer 1 mL of Patent Blue V Sodium Guerbet 2.5%, for the identification of the sentinel lymph node in the left inguinal region, where subsequently lidocaine and ropivacaine hydrochloride were applied. Before Patent Blue V injection, the patient received prophylactic cefazolin and dexamethasone (6 mg IV) according to the institutional perioperative protocol. Several minutes after Patent Blue V injection, the patient developed mild oxygen desaturation, which was managed by deepening anaesthesia and securing the airway with a laryngeal mask. This was followed by progressive severe hypotension that proved refractory to rapid fluid resuscitation. At the onset of cardiovascular collapse, no bronchospasm or early significant cutaneous manifestations were observed. Erythema and piloerection appeared later during the clinical course, while urticaria, angioedema and bronchospasm remained absent throughout the event. These symptoms were promptly treated with intravenous administration of ephedrine and continuous infusion of norepinephrine 0.4 mcg/kg/min, with haemodynamic stabilization of the severe hypotension. Subsequently the patient was intubated on continuous infusion of propofol. Surgical intervention was interrupted after sentinel lymph node biopsy and wide local excision was not performed. The patient was transported to the intensive care unit (ICU) intubated on continuous infusion of norepinephrine 0.5 mg/h, haemodynamically stable, with skin rash and goosebumps. Blood samples, including serum tryptase measurement, were collected during the acute phase of the reaction, within the recommended 1–4-h diagnostic window. A second serum tryptase measurement was obtained 18 h later.

During ICU hospitalization, norepinephrine was gradually de-escalated without any haemodynamic instability. After 14 h of admission to ICU, the patient was extubated, promptly awake, without any neurocognitive deficit and with stable and normal vital parameters. Another serum tryptase dosage was performed at 18 h since ICU admission. First and second results of s-triptase were respectively 16.4 μg/L and 8.2 μg/L (normal range 1–11 μg/L). The increase in s-tryptase is considered significant when its value measured within 1–4 h from the event is 20% basal value +2 μg/L [10].

Following complete clinical recovery, the patient underwent allergological evaluation. The initial assessment included skin and challenge tests for lidocaine, mepivacaine, ropivacaine and articaine, all of which were negative. The patient subsequently underwent wide local excision under local anesthesia without complications. Further allergological investigation included intradermal testing with Patent Blue V Sodium Guerbet 2.5% and cefazolin according to the local Allergy Unit protocol. The patient tested negative for cefazolin and positive for Patent Blue V at an intradermal dilution of 1:100. A positive wheal response compared with the negative control was considered indicative of sensitization.

## 3. Discussion

This case illustrates an atypical presentation of Patent Blue V-induced perioperative anaphylaxis in which severe cardiovascular collapse represented the predominant initial manifestation, whereas cutaneous signs appeared only later during the clinical course. Such delayed cutaneous manifestations may reduce the clinical suspicion of anaphylaxis during the early phase of shock and contribute to delayed recognition [11]. The typical and less common clinical manifestations of Patent Blue V hypersensitivity are summarized in Table 3.

**Table 3:** Clinical manifestations of hypersensitivity reactions to Patent Blue V.

System Involved	Typical Manifestations	Rare Manifestations	Clinical Notes
Cutaneous	Blue wheals, erythema, urticaria, generalized rash	Delayed onset of erythema and piloerection, or isolated piloerection	Absence of skin signs does not exclude anaphylaxis
Cardiovascular	Hypotension, tachycardia	Profound, refractory hypotension, cardiovascular collapse	May represent the only initial manifestation
Respiratory	Bronchospasm, laryngospasm, desaturation	Isolated desaturation without bronchospasm	Often secondary to hypoperfusion
Neurological	Anxiety, confusion	Loss of consciousness	More common in severe reactions
Timing	15–30 min after injection	Immediate (<5 min) or delayed up to 90 min	More severe reactions tend to occur earlier

The main differential diagnoses of acute intraoperative hypotension in this clinical setting are summarized in Table 4. During surgery, the agents most frequently implicated in drug-related allergic reactions are neuromuscular blockers, antibiotics, induction anesthetics, opioids, colloids, NSAIDs, topical antiseptics, and latex [2]. Although latex and antiseptics were not tested by skin-prick methods in this patient, they are unlikely culprits, as the same products were tolerated uneventfully during a subsequent operation.

**Table 4:** Differential diagnosis of acute intraoperative hypotension following Patent Blue V injection.

Diagnosis	Key Features	Elements Against	Diagnostic Clues
PBV-induced anaphylaxis	Sudden hypotension, delayed erythema and piloerection	No specific findings when supported by allergological confirmation	Temporal relationship with PBV injection, elevated serum tryptase, positive intradermal test
Anesthetic-related hypotension	Gradual onset hypotension after induction	Not temporally related to PBV injection	Improvement after fluid administration or anesthetic adjustment
Cefazolin allergy	Similar clinical presentation to anaphylaxis	Negative allergy testing	Negative intradermal test
Vasovagal reaction	Bradycardia with hypotension	Persistent tachycardia and refractory hypotension	Response to atropine
Hemorrhage	Hypotension associated with surgical bleeding	No evidence of significant blood loss	Stable hemoglobin; surgical field inspection
Myocardial ischemia	Hypotension with ECG changes or ventricular dysfunction	No ECG abnormalities	ECG, cardiac biomarkers, echocardiography
Pulmonary embolism	Sudden hypotension, hypoxemia, right ventricular dysfunction	No evidence of acute right ventricular strain	Echocardiography, CT pulmonary angiography
Tension pneumothorax	Sudden hypotension with increased airway pressure	No ventilation abnormalities or increased airway pressure	Clinical examination, lung ultrasound
Excessive anesthetic depth	Progressive hypotension after anesthetic induction	Temporal relationship with PBV injection	Improvement after anesthetic adjustment

Diagnostic tools for suspected PBV hypersensitivity—serum tryptase, skin-prick testing, and intradermal testing—show reported sensitivities of 54%, 80%, and 100%, respectively [12]. In this patient, elevations in tryptase together with a positive intradermal test supported PBV as the causative agent. Although the patient had no documented previous medical exposure to blue dyes, this is consistent with most published cases, which also lack clear prior PBV contact [1,9]. The exact mechanisms underlying Patent Blue V hypersensitivity remain incompletely understood; however, the reaction is thought to result predominantly from IgE-mediated mast-cell activation following prior sensitization, although additional immunological mechanisms have also been proposed.

PBV (also known as E131, disulfide blue, or acid blue 3) is a triphenylmethane dye widely incorporated into numerous consumer and industrial products—foods, textiles, paper, agricultural materials, cosmetics, and medical supplies—which may lead to unrecognized sensitization [13]. Patent Blue V-induced hypersensitivity is an uncommon but well-recognized complication of sentinel lymph node mapping procedures [14].

Incidence of PBV-related anaphylaxis range between 0.1% and 2.8% [5,9,15,16,17], with symptom onset typically occurring 20–30 min after administration, though a broad range from 0 to 90 min has been described [18]. The frequency of severe, life-threatening events is estimated at approximately 0.06% [5,12]. PBV reactions typically involve skin manifestations—such as blue discoloration of the injection area—accompanied by hypotension, while respiratory symptoms are uncommon. Cutaneous discoloration at the injection site with associated hypotension is the classic presentation, whereas respiratory involvement is less common. More severe reactions tend to appear earlier, often within 15–30 min [19,20]. Immediate anaphylaxis is rare [12,21]. In this case, shock developed within the first minutes following Patent Blue V administration, accompanied by refractory hypotension and piloerection. Comparable rapid-onset events have been documented: Šitum et al. described a patient with severe hypotension resistant to epinephrine and ventricular fibrillation [13]; Barthelmes et al. reported several cases occurring within 1–5 min [12]; and Telgenkamp et al. described cardiac arrest shortly after dye administration [22]. The diagnosis of perioperative anaphylaxis was supported by the combination of a clear temporal relationship with Patent Blue V administration, severe refractory hypotension requiring continuous vasopressor support, elevated serum tryptase levels, positive intradermal testing, and the absence of recurrence during subsequent surgery performed without Patent Blue V exposure. Collectively, these findings fulfill the accepted diagnostic framework for perioperative anaphylaxis and make alternative causes of intraoperative cardiovascular collapse highly unlikely.

**Figure 1:**
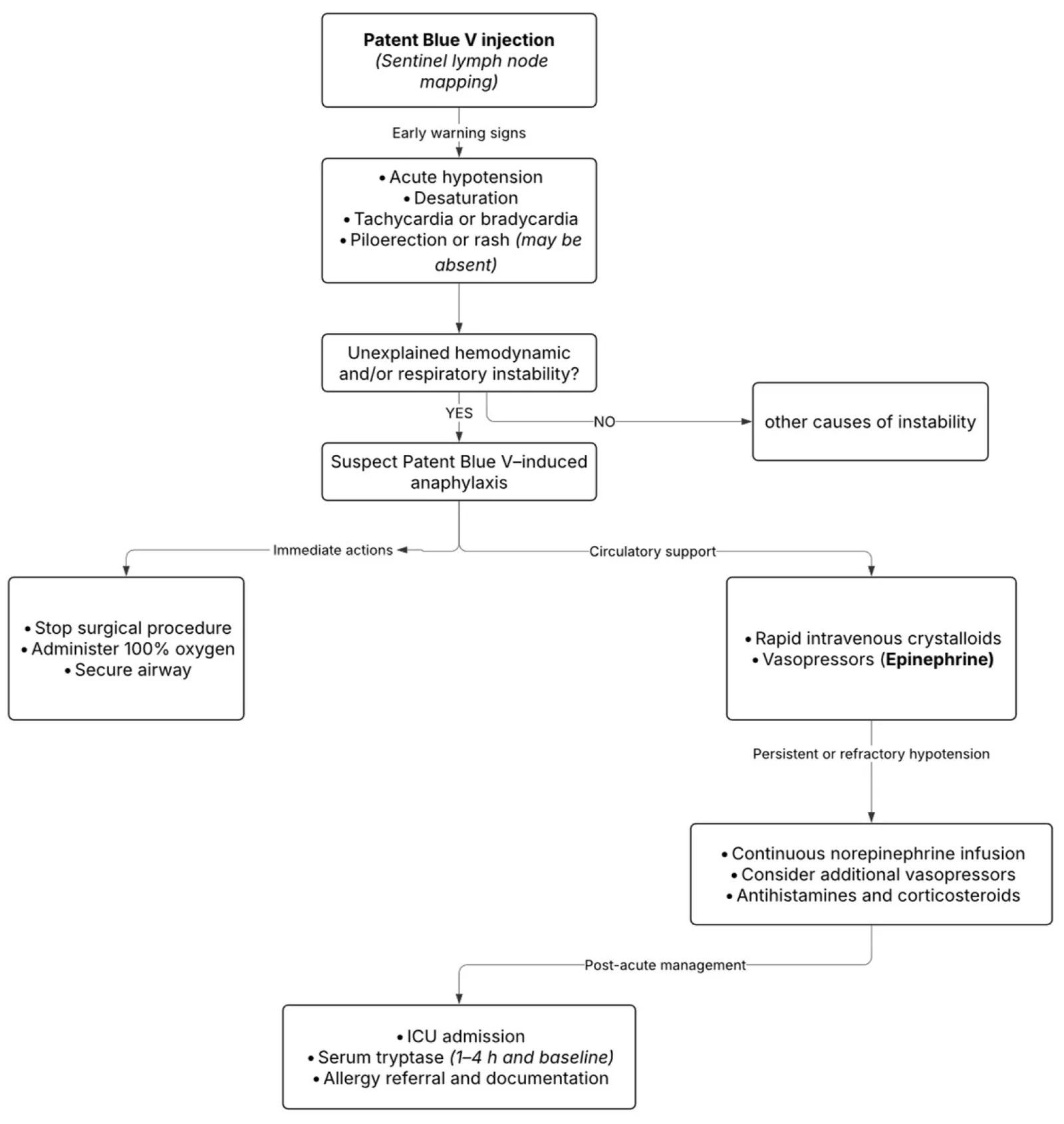
Diagnostic work-up following suspected Patent Blue V-induced perioperative anaphylaxis. The diagram summarizes the sequential diagnostic pathway adopted in the present case, including the acute clinical suspicion, serum tryptase measurements obtained during the acute phase and at follow-up, subsequent allergological evaluation, intradermal testing with cefazolin and Patent Blue V, and the final confirmation of Patent Blue V sensitization. This diagnostic approach supported the identification of Patent Blue V as the causative agent and guided the safe management of subsequent surgical treatment.

Although Patent Blue V-induced anaphylaxis has been previously reported, the present case illustrates several distinctive features. Cardiovascular collapse represented the predominant initial manifestation and preceded the delayed appearance of cutaneous signs. The preoperative administration of dexamethasone may also have contributed to attenuating the initial cutaneous manifestations, although it did not prevent the development of severe cardiovascular collapse. In addition, prolonged vasopressor support was required because of persistent refractory hypotension. The diagnosis was further strengthened by both elevated serum tryptase levels and positive intradermal testing, and by the absence of recurrence during subsequent surgery performed without Patent Blue V exposure [23]. The diagnostic work-up leading to the identification of Patent Blue V as the causative agent is summarized in Figure 1. PBV clearance occurs primarily through the urine over 24–48 h, with a smaller proportion eliminated in bile [9,23]. In our patient, hypotension persisted for hours and required extended norepinephrine support. Although intravenous epinephrine is recommended by current international guidelines as the first-line treatment for perioperative anaphylaxis because of its combined α- and β-adrenergic effects, the present patient initially manifested isolated severe cardiovascular collapse in the absence of bronchospasm or airway edema [24,25,26,27]. Intravenous epinephrine was not administered because the initial clinical presentation was not immediately recognized as perioperative anaphylaxis, and alternative causes of acute intraoperative hypotension were initially considered during the differential diagnostic process. Once the diagnosis became more evident, the patient’s hemodynamic status had progressively improved following aggressive fluid resuscitation, ephedrine administration and continuous norepinephrine infusion. This observation should not be interpreted as suggesting that norepinephrine replaces epinephrine, which remains the recommended first-line treatment according to current international guidelines. Immediate aggressive fluid resuscitation, ephedrine administration, and continuous norepinephrine infusion were therefore initiated to rapidly restore hemodynamic stability. Because persistent refractory hypotension remained the predominant clinical feature, norepinephrine support was progressively escalated, resulting in gradual cardiovascular stabilization. This atypical presentation reinforces the importance of maintaining a high index of suspicion during sentinel lymph node procedures, even when the classical features of anaphylaxis are not shortly evident. Alternative sentinel lymph node mapping techniques, including radioisotope-only mapping, indocyanine green fluorescence and superparamagnetic iron oxide, may represent valuable alternatives in patients with previous Patent Blue V hypersensitivity or in selected high-risk patients.

## 4. Conclusions

Anaphylaxis caused by PBV remains a serious complication that demands careful vigilance [22]. Prompt management—cardiopulmonary resuscitation, adrenergic agents, mast-cell stabilizers, and corticosteroids—is crucial. Vasopressors may have a rapid effect or may necessitate prolonged infusion, and ongoing cardiovascular depression often calls for intensive care management. In patients at elevated risk, prophylactic anti-allergic medications may be considered before PBV injection. To the best of our knowledge, fatalities associated with Patent Blue V-induced anaphylaxis appear to be exceptionally rare in the published literature. Patients undergoing procedures involving PBV should be informed preoperatively of potential adverse reactions. Patent Blue V-induced anaphylaxis, although rare, may initially present with severe cardiovascular collapse before the appearance of delayed and often subtle cutaneous manifestations. Clinicians should therefore be aware that the absence of early skin signs does not exclude perioperative anaphylaxis. High clinical suspicion, prompt recognition, and early hemodynamic support are essential to optimize patient outcomes. This case highlights the importance of maintaining a high index of suspicion for Patent Blue V-induced anaphylaxis in the operating theatre, particularly when cardiovascular collapse occurs without typical cutaneous manifestations.

## Figures and Tables

**Table 1:** Perioperative timeline, hemodynamic parameters and management.

Time	Event	Hemodynamic Parameters	Treatment/Intervention
13:38	Admission to preoperative area	Stable	Preoperative assessment
13:45	Peripheral venous access placed	Stable	Standard monitoring
14:05	Arrival in operating room	SpO_2_ 96% (room air), BP 125/60 mmHg, HR 82 bpm	Baseline assessment
14:15	General anesthesia induction with LMA	SpO_2_ 98%, BP 112/55 mmHg, HR 83 bpm	Propofol infusion initiated; fentanyl 100 mcg
14:25	Administration of dexamethasone and cefazolin	SpO_2_ 98%, BP 109/56 mmHg, HR 84 bpm	Dexamethasone 6 mg IV; cefazolin 2 g IV
14:25	Patent Blue V injection	Stable	Patent Blue V 1 mL SC
14:26	Local anesthetic infiltration	SpO_2_ 98%, BP 105/54 mmHg, HR 86 bpm	Ropivacaine 100 mg + lidocaine 10 mg
14:35	Initial deterioration	SpO_2_ 90%, BP 98/50 mmHg, HR 98 bpm	Increased monitoring
14:45	Severe hypotension	BP 70/40 mmHg, HR 102 bpm	Ephedrine boluses; calcium chloride 1 g IV
14:50	Refractory hypotension	BP 62/40 mmHg, HR 107 bpm	Norepinephrine started 0.2 mcg/kg/min
14:50	Suspected anaphylaxis	BP 75/50 mmHg, HR 105 bpm	Norepinephrine increased to 0.4 mcg/kg/min
14:55	Cutaneous signs	Improving	Arterial catheter; erythema and piloerection
15:05	Orotracheal intubation	BP 98/58 mmHg, HR 102 bpm, SpO_2_ 98%	Rocuronium, fentanyl, midazolam
15:15	Clinical stabilization	BP 108/54 mmHg, HR 90 bpm	Central venous catheter; surgery interrupted
15:35	Transfer to ICU	BP 109/58 mmHg, HR 89 bpm	ICU admission
ICU stay	Progressive improvement	Stable	Gradual norepinephrine tapering
ICU stay	Diagnostic evaluation	—	Serum tryptase 16.4 μg/L
14 h later	Extubation	Stable	Full recovery
Follow-up	Repeat tryptase	—	8.2 μg/L
Post-recovery allergological evaluation	Allergy work-up	—	Negative for cefazolin/local anesthetics
Follow-up	Intradermal testing	—	Positive for Patent Blue V
Subsequent surgery	Wide local excision	Stable	No complications

**Table 2:** Drugs administered during perioperative management.

Drug	Dose	Route	Indication
Patent Blue V	1 mL	Subcutaneous	Sentinel lymph node mapping
Lidocaine	10 mg	Local infiltration	Local anesthesia
Ropivacaine	100 mg	Local infiltration	Local anesthesia
Cefazolin	2 g	IV	Surgical prophylaxis
Dexamethasone	6 mg	IV	Perioperative prophylaxis
Propofol	TCI 2–4 mcg/mL	IV infusion	General anesthesia
Fentanyl	200 mcg total	IV	Analgesia/anesthesia
Ephedrine	Repeated 10 mg boluses	IV	Severe hypotension
Calcium chloride	2 g total	IV	Refractory hypotension
Hydrocortisone	200 mg	IV	Suspected anaphylaxis
Norepinephrine	Up to 0.4 mcg/kg/min	IV infusion	Refractory shock
Rocuronium	80 mg	IV	Intubation
Midazolam	5 mg	IV	Sedation
Pantoprazole	40 mg	IV	ICU management
Isolyte	80 mL/h	IV infusion	Maintenance therapy
ER III	1500 mL	IV	Volume expansion

## Data Availability

The data supporting the findings of this case report are available from the corresponding author upon reasonable request, subject to institutional regulations and patient privacy requirements. CARE Guidelines: This case report has been prepared in accordance with the CARE (CAse REport) reporting guidelines.
